# 2-(4-Chloro­phen­yl)chromen-4-one

**DOI:** 10.1107/S1600536811043832

**Published:** 2011-11-05

**Authors:** Shailja Singh, Manavendra K. Singh, Alka Agarwal, Satish K. Awasthi

**Affiliations:** a Chemical Biology Laboratory, Department of Chemistry, University of Delhi 110 007, India; b Department of Medicinal Chemistry, Institute of Medical Sciences, Banaras Hindu University, Varanasi 225 001 UP, India

## Abstract

The title compound, C_15_H_9_ClO_2_, is a synthetic flavonoid obtained by the cyclization of 3-(4-chloro­phen­yl)-1-(2-hy­droxy­phen­yl)prop-2-en-1-one. The 4-chloro­phenyl ring is twisted at an angle of 11.54° with respect to the chromen-4-one skeleton. In the crystal, pairs of mol­ecules are inter­connected by weak Cl⋯Cl inter­actions [3.3089 (10) Å] forming dimmers which are further peripherally connected through inter­molecular C—H⋯O hydrogen bonds.

## Related literature

For general features and crystal structures of flavanoids, see: Tim Cushnie & Lamb (2005 ▶); Wera *et al.* (2011 ▶). For crystal structures of small mol­ecules, see: Singh, Agarwal & Awasthi (2011 ▶); Singh, Singh *et al.* (2011 ▶). For the synthesis, see: Migrdichian (1957 ▶); Awasthi *et al.* (2009 ▶); Shah *et al.* (1955 ▶). For inter­molecular inter­actions and bond lengths and angles, see: Reddy *et al.* (2006 ▶); Wang *et al.* (2010 ▶); Desiraju & Steiner (1999 ▶); Waller *et al.* (2003 ▶); Allen *et al.* (1987 ▶).
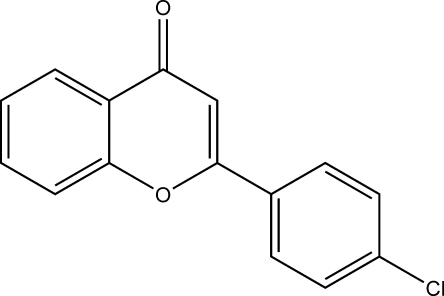


## Experimental

### 

#### Crystal data

C_15_H_9_ClO_2_
*M* *_r_* = 256.67
Monoclinic, 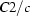

*a* = 22.1564 (16) Å
*b* = 3.8745 (2) Å
*c* = 26.7728 (18) Å
β = 95.524 (6)°
*V* = 2287.6 (3) Å^3^
*Z* = 8
Mo *K*α radiation
μ = 0.32 mm^−1^
*T* = 293 K
0.40 × 0.39 × 0.38 mm

#### Data collection

Oxford Xcalibur Eos diffractometer
Absorption correction: multi-scan (*CrysAlis PRO*; Oxford Diffraction, 2009 ▶) *T*_min_ = 0.938, *T*_max_ = 0.941
8152 measured reflections
2249 independent reflections
1910 reflections with *I* > 2σ(*I*)
*R*_int_ = 0.037
Standard reflections: 0

#### Refinement

*R*[*F* ^2^ > 2σ(*F* ^2^)] = 0.049
*wR*(*F* ^2^) = 0.119
*S* = 1.10
2249 reflections
163 parameters
H-atom parameters constrained
Δρ_max_ = 0.20 e Å^−3^
Δρ_min_ = −0.24 e Å^−3^

### 

Data collection: *CrysAlis PRO* (Oxford Diffraction, 2009 ▶); cell refinement: *CrysAlis PRO*; data reduction: *CrysAlis PRO*; program(s) used to solve structure: *SHELXS97* (Sheldrick, 2008 ▶); program(s) used to refine structure: *SHELXL97* (Sheldrick, 2008 ▶); molecular graphics: *Mercury* (Macrae *et al.*, 2008 ▶); software used to prepare material for publication: *publCIF* (Westrip, 2010 ▶).

## Supplementary Material

Crystal structure: contains datablock(s) I, global. DOI: 10.1107/S1600536811043832/zj2023sup1.cif

Structure factors: contains datablock(s) I. DOI: 10.1107/S1600536811043832/zj2023Isup2.hkl

Supplementary material file. DOI: 10.1107/S1600536811043832/zj2023Isup3.cml

Additional supplementary materials:  crystallographic information; 3D view; checkCIF report

## Figures and Tables

**Table 1:** Hydrogen-bond geometry (Å, °)

*D*—H⋯*A*	*D*—H	H⋯*A*	*D*⋯*A*	*D*—H⋯*A*
C11—H11⋯O2^i^	0.93	2.64	3.345 (3)	134 (1)

Symmetry code: (i) 
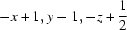
.

**Fig. 1.**
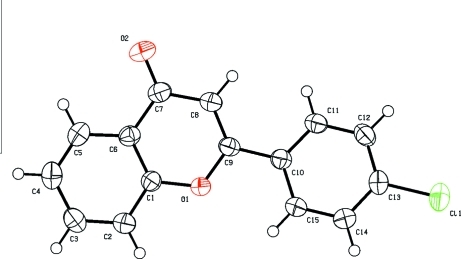
ORTEP diagram of the molecule with thermal ellipsoids drawn at 50% probability level Color code: White: C; red: O; green: Cl; white: H.

**Fig. 2.**
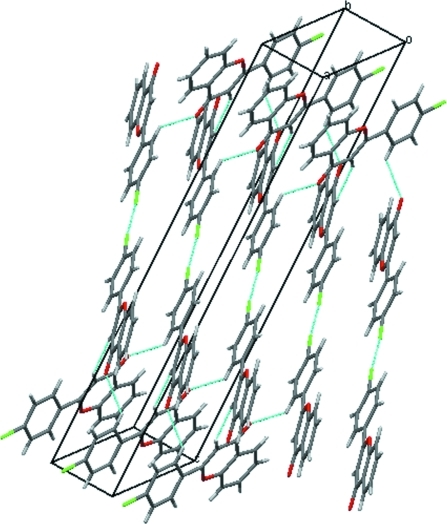
Packing diagram of molecule showing centroid–centroid distance between two parallel lying cromone ring and intermolecular hydrogen bonding.

## supplementary crystallographic information

### Comment

The term flavonoid generally includes a group of natural products containing a C6—C3—C6 carbon skeleton or more specifically phenylbenzopyran functionality in the molecule. Flavones (*flavus* = yellow), a class of the flavonoids mainly found in cereals and herbs. Flavanoids exhibit a wide range of biological activities such as antibacterial, anti-inflammatory, antioxidants, antifungal, antitumour and antimalarial (Tim Cushnie & Lamb 2005). Recently, few flavanoids have also been characterized in solid state (Wera *et al.*, 2011). Continuing our ongoing work on antimalarials (Awasthi *et al.*, 2009) and crystal structure of small molecules (Singh, Agarwal & Awasthi, 2011; Singh, Singh *et al.*, 2011), here we wish to report the crystal structure of 2-(4-chlorophenyl)chromen-4-one.

In the title compound (Fig. 1),the bond lengths and bond angles are usual and are comparable with the analogues structure of 2-phenyl-4*H*-chromen-4-one (flavone) reported earlier (Allen *et al.*, 1987; Waller *et al.*, 2003). The 4-chlorophenyl ring in the molecule is twisted at an angle of 11.54° relative to the chromen-4-one skeleton confirming nearly planner structure. The centroid–centroid distance between two parallel chromone ring in the molecule is 3.87 Å. Further, it is evident from the crystal packing structure (Fig. 2) that 8 molecules are present in a unit cell and adjacent chromone units are parallel in a given column, thus forming a herringbone type pattern. Moreover,crystal packing in the molecule is stabilized by weaker intermolecular hydrogen bonding C11—H11—O2 [D = 3.34 (3) Å] which is very well supported by earlier findings (Desiraju & Steiner, 1999). Further, weak interaction among atoms in molecule such as Cl1—Cl1 (*x*, -1 + *y*, 1/2 - *z*) [3.30 Å] (Reddy *et al.*,2006) and C8—H8—H8—C8 [2.26 Å](Wang *et al.*, 2010) are also responsible for stability in the crystal packing. Further, intermolecular Cl1—Cl1 short interaction forms a dimeric unit which are further peripherically links to six other molecules through C—H—O and C—H—H—C interactions.

### Experimental

The synthesis of the title compound was carried out in two steps according to the published procedure. (Migrdichian 1957; Awasthi *et al.*, 2009). In the first step, an aqueous solution of sodium hydroxide (10% *w*/*v*, 10 ml) was added to a solution of 2-hydroxyacetophenone (1.77 g m, 10 mmol) and 4-chlorobenzaldehyde (1.73 g m, 10 mmol) in minimum amount of methanol (3–5 ml) at ice cooled flask. The reaction mixture was allowed to draw closer to room temperature and stirred for 18–20 h yielded a yellow solid. The completion of the reaction was monitored by thin layer chromatography. After completion of the reaction, the mixture was neutralized with 10% hydrochloric acid in water. The compound was characterized by ^1^H NMR, ^13^C NMR, FT–IR and EI–MS.

In second step, the cyclization was carried out according to published procedure (Shah *et al.*, 1955). Briefly, 3-(4-chlorophenyl)-1-(2-hydroxyphenyl)propenone (40 mg, 0.12 mmol) & SeO_2_ (39 mg, 0.35 mmol) were added to dry amyl alcohol (30 ml) and the mixture was heated in an oil bath at 140–150 °C so that the entire compound was completely dissolved in the solvent. The reaction mixture was refluxed for 12 h and completion of the reaction was monitored by TLC. The reaction mixture was then filtered and dried in vacuum and purified by silica gel column using (Pet. ether: EtOAc, 2:3) as eluent. The recrystalliation of an isolated compound from PE/ethylacetate to afford 2-(4-chlorophenyl)chromen-4-one (10 mg, 20.1%) as white solid, m.p 177–178°C. *R*_f_ 0.6 (PE; EtOAc, 2:3). FT–IR ν_max_ (KBr) cm^-1^: 1651 (C═O), 1606 and 1510 (C═C aromatic), 1263 (C—O); ^1^H NMR (300 Mz, CDCl_3_) p.p.m.: δ 6.63 (1*H*, s, H-3, pyrone ring), δ 7.32–7.48 (4*H*, m, Ar-H, H'-5, H'-6, H'-7, H'-8), 7.20 (2*H*, dd, J = 2.4 Hz, H'-5, H'-3), 7.28 (2*H*, dd, J = 2.1 Hz, H'-2, H'-6), ^13^C NMR (300 Mz, CDCl_3_) ppm: EI–MS: m/*z* 255 [*M*+].

For crystallization 5 mg of compound dissolved in 5 ml mixture of Petroleum ether/ethylacetate (80:20) and left for several days at ambient temperature which yielded fine needle shape crystals.

### Refinement

All H atoms were located from Fourier map (range of C—H = 0.93 Å) allowed to refine freely.

### Crystal data

**Table:**

C_15_H_9_ClO_2_	*F*(000) = 528
*M**_r_* = 256.67	*D*_x_ = 1.490 Mg m^−^^3^
Monoclinic, *C*2/*c*	Mo *K*α radiation, λ = 0.71073 Å
Hall symbol: -C 2yc	Cell parameters from 3949 reflections
*a* = 22.1564 (16) Å	θ = 3.1–32.6°
*b* = 3.8745 (2) Å	µ = 0.32 mm^−^^1^
*c* = 26.7728 (18) Å	*T* = 293 K
β = 95.524 (6)°	Needle, colourless
*V* = 2287.6 (3) Å^3^	0.40 × 0.39 × 0.38 mm
*Z* = 8	

### Data collection

**Table:**

Oxford Xcalibur Eos diffractometer	2249 independent reflections
Radiation source: fine-focus sealed tube	1910 reflections with *I* > 2σ(*I*)
graphite	*R*_int_ = 0.037
ω scans	θ_max_ = 26.0°, θ_min_ = 3.4°
Absorption correction: multi-scan (*CrysAlis PRO*; Oxford Diffraction, 2009)	*h* = −26→26
*T*_min_ = 0.938, *T*_max_ = 0.941	*k* = −4→4
8152 measured reflections	*l* = −33→33

### Refinement

**Table:**

Refinement on *F*^2^	Primary atom site location: structure-invariant direct methods
Least-squares matrix: full	Secondary atom site location: difference Fourier map
*R*[*F*^2^ > 2σ(*F*^2^)] = 0.049	Hydrogen site location: inferred from neighbouring sites
*wR*(*F*^2^) = 0.119	H-atom parameters constrained
*S* = 1.10	*w* = 1/[σ^2^(*F*_o_^2^) + (0.0483*P*)^2^ + 1.836*P*] where *P* = (*F*_o_^2^ + 2*F*_c_^2^)/3
2249 reflections	(Δ/σ)_max_ = 0.009
163 parameters	Δρ_max_ = 0.20 e Å^−^^3^
0 restraints	Δρ_min_ = −0.24 e Å^−^^3^

### Special details

**Table:**

Geometry. All e.s.d.'s (except the e.s.d. in the dihedral angle between two l.s. planes) are estimated using the full covariance matrix. The cell e.s.d.'s are taken into account individually in the estimation of e.s.d.'s in distances, angles and torsion angles; correlations between e.s.d.'s in cell parameters are only used when they are defined by crystal symmetry. An approximate (isotropic) treatment of cell e.s.d.'s is used for estimating e.s.d.'s involving l.s. planes.
Refinement. Refinement of *F*^2^ against ALL reflections. The weighted *R*-factor *wR* and goodness of fit *S* are based on *F*^2^, conventional *R*-factors *R* are based on *F*, with *F* set to zero for negative *F*^2^. The threshold expression of *F*^2^ > σ(*F*^2^) is used only for calculating *R*-factors(gt) *etc*. and is not relevant to the choice of reflections for refinement. *R*-factors based on *F*^2^ are statistically about twice as large as those based on *F*, and *R*-factors based on ALL data will be even larger.

### Fractional atomic coordinates and isotropic or equivalent isotropic displacement parameters (Å^2^)

**Table:**

	*x*	*y*	*z*	*U*_iso_*/*U*_eq_	
Cl1	0.31934 (3)	0.85487 (18)	0.02358 (2)	0.0570 (2)	
C9	0.53822 (9)	1.4266 (6)	0.14239 (7)	0.0337 (5)	
C6	0.64493 (9)	1.7175 (6)	0.18541 (7)	0.0354 (5)	
C10	0.48486 (9)	1.2780 (6)	0.11311 (7)	0.0337 (5)	
C1	0.63679 (9)	1.6341 (5)	0.13486 (7)	0.0339 (5)	
C13	0.38335 (9)	1.0144 (6)	0.05827 (8)	0.0397 (5)	
C15	0.48053 (10)	1.2743 (6)	0.06106 (8)	0.0400 (5)	
H15	0.5123	1.3606	0.0445	0.048*	
C8	0.54262 (10)	1.5012 (7)	0.19128 (8)	0.0461 (6)	
H8	0.5095	1.4573	0.2092	0.055*	
C5	0.70028 (10)	1.8653 (6)	0.20370 (8)	0.0428 (5)	
H5	0.7069	1.9248	0.2374	0.051*	
C2	0.68118 (10)	1.6940 (6)	0.10315 (8)	0.0431 (5)	
H2	0.6748	1.6370	0.0693	0.052*	
C14	0.42994 (10)	1.1448 (6)	0.03366 (8)	0.0436 (6)	
H14	0.4273	1.1455	−0.0012	0.052*	
C11	0.43729 (10)	1.1410 (6)	0.13715 (8)	0.0416 (5)	
H11	0.4397	1.1385	0.1720	0.050*	
C12	0.38674 (10)	1.0092 (6)	0.10974 (8)	0.0431 (5)	
H12	0.3551	0.9172	0.1260	0.052*	
C7	0.59653 (11)	1.6468 (6)	0.21751 (8)	0.0445 (6)	
O2	0.60167 (9)	1.7078 (6)	0.26277 (6)	0.0690 (6)	
C3	0.73488 (10)	1.8388 (6)	0.12232 (9)	0.0472 (6)	
H3	0.7651	1.8809	0.1013	0.057*	
C4	0.74463 (11)	1.9233 (6)	0.17286 (9)	0.0475 (6)	
H4	0.7814	2.0193	0.1856	0.057*	
O1	0.58414 (6)	1.4868 (4)	0.11337 (5)	0.0382 (4)	

### Atomic displacement parameters (Å^2^)

**Table:**

	*U*^11^	*U*^22^	*U*^33^	*U*^12^	*U*^13^	*U*^23^
Cl1	0.0454 (4)	0.0656 (5)	0.0582 (4)	−0.0157 (3)	−0.0049 (3)	−0.0043 (3)
C9	0.0323 (10)	0.0380 (12)	0.0317 (10)	0.0045 (9)	0.0076 (8)	0.0045 (9)
C6	0.0365 (11)	0.0363 (12)	0.0332 (11)	0.0054 (9)	0.0013 (9)	0.0023 (9)
C10	0.0323 (11)	0.0353 (11)	0.0337 (10)	0.0045 (9)	0.0033 (8)	0.0035 (9)
C1	0.0319 (10)	0.0358 (12)	0.0336 (10)	0.0005 (9)	0.0011 (8)	0.0027 (9)
C13	0.0326 (11)	0.0390 (12)	0.0466 (12)	−0.0030 (10)	−0.0011 (9)	−0.0010 (10)
C15	0.0375 (11)	0.0492 (13)	0.0342 (11)	−0.0041 (10)	0.0082 (9)	0.0025 (10)
C8	0.0389 (12)	0.0672 (16)	0.0331 (11)	−0.0059 (11)	0.0085 (9)	0.0006 (11)
C5	0.0437 (12)	0.0432 (13)	0.0398 (12)	0.0003 (11)	−0.0045 (10)	−0.0018 (10)
C2	0.0408 (12)	0.0529 (14)	0.0361 (11)	−0.0015 (11)	0.0060 (9)	−0.0002 (10)
C14	0.0444 (13)	0.0533 (15)	0.0331 (11)	−0.0050 (11)	0.0038 (10)	−0.0002 (10)
C11	0.0393 (12)	0.0529 (14)	0.0334 (11)	−0.0009 (11)	0.0076 (9)	0.0040 (10)
C12	0.0347 (11)	0.0496 (14)	0.0461 (12)	−0.0054 (10)	0.0103 (10)	0.0052 (11)
C7	0.0451 (13)	0.0563 (15)	0.0321 (11)	0.0007 (12)	0.0038 (9)	−0.0031 (10)
O2	0.0648 (12)	0.1114 (17)	0.0314 (9)	−0.0159 (11)	0.0068 (8)	−0.0149 (10)
C3	0.0379 (12)	0.0508 (15)	0.0539 (14)	−0.0036 (11)	0.0105 (10)	0.0053 (12)
C4	0.0382 (12)	0.0450 (14)	0.0574 (14)	−0.0045 (11)	−0.0045 (11)	0.0018 (12)
O1	0.0326 (7)	0.0541 (10)	0.0282 (7)	−0.0041 (7)	0.0045 (6)	−0.0028 (7)

### Geometric parameters (Å, °)

**Table:**

Cl1—C13	1.733 (2)	C8—C7	1.441 (3)
C9—C8	1.335 (3)	C8—H8	0.9300
C9—O1	1.358 (2)	C5—C4	1.362 (3)
C9—C10	1.471 (3)	C5—H5	0.9300
C6—C1	1.386 (3)	C2—C3	1.370 (3)
C6—C5	1.399 (3)	C2—H2	0.9300
C6—C7	1.463 (3)	C14—H14	0.9300
C10—C15	1.388 (3)	C11—C12	1.377 (3)
C10—C11	1.392 (3)	C11—H11	0.9300
C1—O1	1.374 (2)	C12—H12	0.9300
C1—C2	1.379 (3)	C7—O2	1.229 (3)
C13—C14	1.373 (3)	C3—C4	1.389 (3)
C13—C12	1.373 (3)	C3—H3	0.9300
C15—C14	1.374 (3)	C4—H4	0.9300
C15—H15	0.9300		
			
C8—C9—O1	122.4 (2)	C6—C5—H5	119.5
C8—C9—C10	125.9 (2)	C3—C2—C1	118.9 (2)
O1—C9—C10	111.72 (17)	C3—C2—H2	120.5
C1—C6—C5	117.7 (2)	C1—C2—H2	120.5
C1—C6—C7	119.74 (19)	C13—C14—C15	119.4 (2)
C5—C6—C7	122.57 (19)	C13—C14—H14	120.3
C15—C10—C11	118.6 (2)	C15—C14—H14	120.3
C15—C10—C9	120.89 (19)	C12—C11—C10	120.5 (2)
C11—C10—C9	120.54 (18)	C12—C11—H11	119.7
O1—C1—C2	116.05 (18)	C10—C11—H11	119.7
O1—C1—C6	122.07 (18)	C13—C12—C11	119.5 (2)
C2—C1—C6	121.9 (2)	C13—C12—H12	120.2
C14—C13—C12	121.1 (2)	C11—C12—H12	120.2
C14—C13—Cl1	119.24 (17)	O2—C7—C8	123.3 (2)
C12—C13—Cl1	119.70 (17)	O2—C7—C6	122.7 (2)
C14—C15—C10	120.9 (2)	C8—C7—C6	114.01 (18)
C14—C15—H15	119.5	C2—C3—C4	120.6 (2)
C10—C15—H15	119.5	C2—C3—H3	119.7
C9—C8—C7	122.8 (2)	C4—C3—H3	119.7
C9—C8—H8	118.6	C5—C4—C3	119.9 (2)
C7—C8—H8	118.6	C5—C4—H4	120.1
C4—C5—C6	121.0 (2)	C3—C4—H4	120.1
C4—C5—H5	119.5	C9—O1—C1	118.96 (15)
			
C8—C9—C10—C15	167.0 (2)	C15—C10—C11—C12	−0.9 (3)
O1—C9—C10—C15	−12.2 (3)	C9—C10—C11—C12	178.5 (2)
C8—C9—C10—C11	−12.3 (4)	C14—C13—C12—C11	0.9 (4)
O1—C9—C10—C11	168.48 (19)	Cl1—C13—C12—C11	−179.02 (18)
C5—C6—C1—O1	179.74 (19)	C10—C11—C12—C13	−0.2 (4)
C7—C6—C1—O1	0.3 (3)	C9—C8—C7—O2	−178.0 (3)
C5—C6—C1—C2	−0.2 (3)	C9—C8—C7—C6	2.2 (4)
C7—C6—C1—C2	−179.6 (2)	C1—C6—C7—O2	178.3 (2)
C11—C10—C15—C14	1.3 (4)	C5—C6—C7—O2	−1.1 (4)
C9—C10—C15—C14	−178.1 (2)	C1—C6—C7—C8	−1.9 (3)
O1—C9—C8—C7	−0.9 (4)	C5—C6—C7—C8	178.7 (2)
C10—C9—C8—C7	180.0 (2)	C1—C2—C3—C4	0.2 (4)
C1—C6—C5—C4	−0.3 (3)	C6—C5—C4—C3	0.7 (4)
C7—C6—C5—C4	179.1 (2)	C2—C3—C4—C5	−0.7 (4)
O1—C1—C2—C3	−179.7 (2)	C8—C9—O1—C1	−0.9 (3)
C6—C1—C2—C3	0.3 (3)	C10—C9—O1—C1	178.32 (17)
C12—C13—C14—C15	−0.5 (4)	C2—C1—O1—C9	−178.86 (19)
Cl1—C13—C14—C15	179.43 (18)	C6—C1—O1—C9	1.2 (3)
C10—C15—C14—C13	−0.6 (4)		

### Hydrogen-bond geometry (Å, °)

**Table:**

*D*—H···*A*	*D*—H	H···*A*	*D*···*A*	*D*—H···*A*
C11—H11···O2^i^	0.93	2.64	3.345 (3)	134.(1)

Symmetry codes: (i) −*x*+1, *y*−1, −*z*+1/2.
